# ASSESSING THE EFFECTS OF VR URBAN GREENNESS WITH PEER SUPPORT MODEL FOR MENTAL HEALTH IN AGING POPULATION

**DOI:** 10.1093/geroni/igae098.4263

**Published:** 2024-12-31

**Authors:** Shiyu Lu, Hiu-kwan Chui, Linyan Li

**Affiliations:** The University of Hong Kong, Hong Kong, China (People’s Republic); The University of Hong Kong, Hong Kong, China (People’s Republic); City University of Hong Kong, Hong Kong, China (People’s Republic)

## Abstract

Engaging with green spaces has shown to benefit mental health in later years. However, global urbanization has limited city-dwellers’ access to nature, especially older adults who may struggle to reach such spaces. Virtual Reality (VR) urban greenness offers a cost-effective solution for promoting mental well-being in this demographic. This study aimed to assess the effectiveness of the VR urban greenness with peer support model in enhancing mental health among the aging population. From October 2023 to July 2024, we collaboratively developed a 5-minute 360-degree VR video of urban green spaces with older individuals in Hong Kong, using the photovice method. Subsequently, we conducted a 3-arm randomized controlled trial involving 25 participants with mild depressive symptoms in each arm: VR urban green space with peer supporters (A), VR urban green space without peer supporters (B), and non-biophilic VR intervention without peer supporters as a control group. Both intervention groups (A and B) reported significant reductions in stress levels, anxiety, and negative emotions, coupled with an increase in positive emotions. Conversely, no significant changes were observed in the control group. Following the qualitative study, it emerged that participants in both intervention groups displayed favorable attitudes towards VR and showed strong acceptance of the 360-degree VR urban green spaces. The research further delved into the policy and practical implications of integrating VR technology into mental health promotion strategies for the elderly.

